# Selection and Validation of Reference Genes for RT-qPCR Across Different Organs, Flower Bud Developmental Stages, and Hormone Treatments in *Ilex verticillata*

**DOI:** 10.3390/plants15193001

**Published:** 2026-09-30

**Authors:** Ying Yang, Zhonghua Wang, Huwei Yuan, Bingsong Zheng, Jinjian Yu, Yi He, Dongbin Xu

**Affiliations:** 1 School of Modern Agriculture, Zhejiang Wanli University, Ningbo 315100, China; yingyang@zwu.edu.cn (Y.Y.); wang1972@zwu.edu.cn (Z.W.); 2 State Key Laboratory for Development and Utilization of Forest Food Resources, Zhejiang Agriculture and Forestry University, Hangzhou 311300, China; hwyuan@zafu.edu.cn (H.Y.); bszheng@zafu.edu.cn (B.Z.); 3 Ningbo Key Laboratory of Ornamental Plant Genetics, Breeding and Utilization, Ningbo City College of Vocational Technology, Ningbo 315100, China; 4 Ningbo Forestry Development Center, Ningbo 315100, China

**Keywords:** *Ilex verticillata*, reference gene, RT-qPCR, flower buds, hormonal treatment

## Abstract

Gene expression profiling plays a pivotal role in elucidating molecular regulatory networks and identifying functionally critical genes. However, the accuracy and reliability of quantitative real-time PCR (qPCR) assays fundamentally depend on the selection of stable expressed reference genes. *Ilex verticillata*, an economically important ornamental shrub, remains poorly characterized at the molecular level. In this study, the expression stability of 15 candidate reference genes was systematically evaluated across four major tissue types—roots, stems, leaves, and fruits. Using raw *C_t_* values, the stability of each candidate reference gene was rigorously assessed and ranked by four widely accepted algorithms: geNorm, NormFinder, BestKeeper, and RefFinder. Based on consensus rankings, six genes (*18S*, *28S*, *Actin2*, *EIF4A*, *PT*, and *PP2A*) were preliminarily identified as the most stable candidates. These six genes were subsequently validated in two additional experimental contexts: flower buds collected at distinct developmental stages and leaves subjected to a panel of phytohormone treatments. Results revealed that *Actin2* exhibited the highest overall expression stability across all tested conditions, with *28S* serving as a robust alternative; in contrast, *18S* displayed comparatively low stability. To further corroborate the suitability of the selected reference genes, three biologically relevant target genes —*IvFT*, *IvAGL19*, and *IvWUS2*—were chosen for validation. Normalization using either the top-ranked single reference gene *Actin2* or the optimal multi-gene combination yielded highly consistent and biologically plausible expression profiles for all three targets. Conversely, normalization against the least stable reference gene produced markedly inconsistent and unreliable expression patterns. Collectively, these findings establish a rigorous, experimentally validated framework for reference gene selection in qPCR-based studies of flower bud development and hormonal responses in *Ilex verticillata*.

## 1. Introduction

*Ilex verticillata*, commonly known as North American winterberry, is a deciduous shrub of the genus *Ilex* in the family Aquifoliaceae. Native to North America, this species was first introduced to China in 2006 and has since become a highly valued ornamental plant, characterized by its deciduous habit and striking winter fruit [1]. It is widely used as a potted plant, cut branch, floral material, and landscape nursery stock, thereby increasing farmers’ incomes and supporting local economic development [2,3]. To date, most research on *Ilex verticillata* has primarily focused on conventional breeding practices and physiological–biochemical characterization, whereas investigations into cultivar improvement and underlying molecular mechanisms are still in their early stages [4,5,6]. Therefore, it is important to study gene function and expression patterns during the developmental stages of this species, and suitable reference genes are essential for gene expression analysis in *Ilex verticillata*.

Gene expression analysis serves as a powerful tool for unraveling molecular regulatory mechanisms and identifying key genes [7]. Real-time quantitative polymerase chain reaction (RT-qPCR) is a widely used method for studying gene expression, offering advantages such as high sensitivity, strong specificity, low cost, and operational simplicity [8,9]. However, the accuracy and reliability of RT-qPCR results can be compromised by multiple factors, including RNA integrity, purity, cDNA quality, and reverse transcription efficiency, as well as the choice of reference genes [10]. Generally, reference genes are endogenous genes used to normalize target gene expression data, and they are expected to remain stably expressed under specific experimental conditions [11]. The absence of suitable reference genes may lead to biased gene expression profiles and poor reproducibility. An ideal reference gene should therefore exhibit stable expression independent of cell type, tissue, developmental stage, or growth conditions. Commonly used reference genes in plants include *18S* (18S ribosomal RNA), *GAPDH* (glyceraldehyde-3-phosphate dehydrogenase), *EF-1α* (elongation factor-1-alpha), *TUA* (alpha-tubulin), *TUB* (tubulin), *UBQ* (ubiquitin), and *ACT* (actin) [12,13]. Nevertheless, no single reference gene is universally stable across all conditions. The suitability of a particular reference gene depends on the experimental context, and the use of unstably expressed reference genes may lead to biased results and false positives [14]. Therefore, selecting appropriate reference genes is essential for obtaining accurate and reproducible quantitative data.

In this study, 15 candidate reference genes (*18S*, *28S*, *CYP*, *DNAJ*, *EF-1α*, *Hsp*, *EIF4A*, *PP2A*, *PT*, *TUA*, *TUB*, *UBC*, *UBQ*, *Actin2* and *Actin7*) were selected based on the transcriptome data of *Ilex verticillata* and previously reported literature. A series of algorithms, including geNorm, NormFinder, BestKeeper, and RefFinder, were used to evaluate the expression stability of these candidate genes in different organs of *Ilex verticillata* from multiple statistical perspectives, which led to the preliminary screening of six stably expressed reference genes (*18S*, *28S*, *Actin2*, *EIF4A*, *PT*, *PP2A*). These six reference genes were further examined for their expression stability in flower buds at different developmental stages, as well as in leaves treated with various hormones. To verify the reliability of the selected reference genes, the expression levels of *IvFT*, *IvAGL19*, and *IvWUS2* genes—key regulators of plant flower bud differentiation—were evaluated using the candidate reference genes [15,16,17]. Collectively, these analyses successfully identified reference genes that are stably expressed during the development of *Ilex verticillata,* providing a solid foundation for subsequent studies on molecular mechanisms underlying the growth and development of this species.

## 2. Results

### 2.1. Primer Specificity Analysis

**Figure 1:**
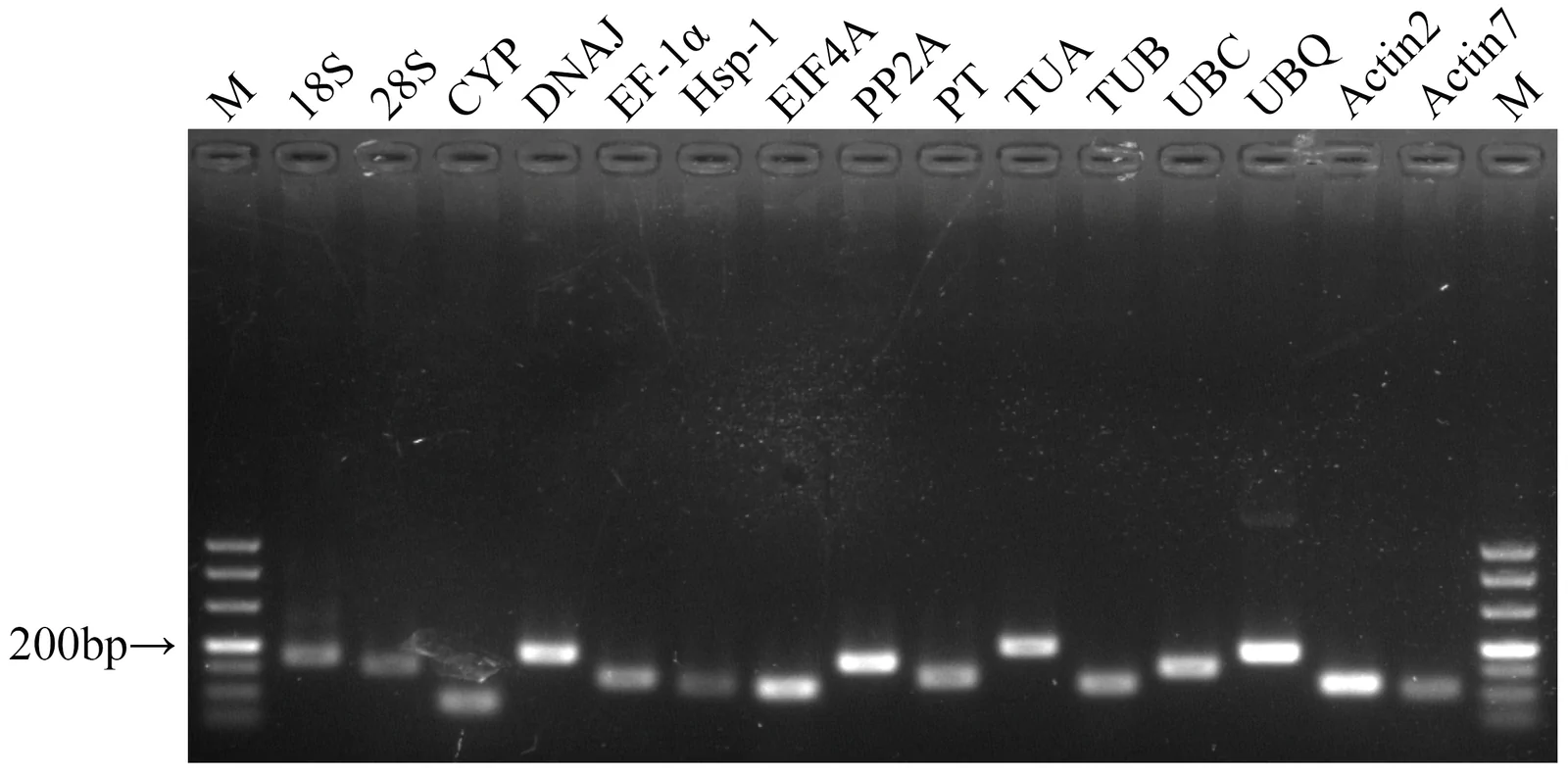
Agarose gel electrophoresis analysis of the specificity of primers for candidate housekeeping genes.

Total RNA was isolated from roots, stems, and leaves of *Ilex verticillata* and reverse-transcribed into cDNA. Following PCR amplification, the products were separated by 1.5% agarose gel electrophoresis. All 15 candidate reference genes yielded single bands of the expected sizes (Figure 1), without primer dimers or non-specific amplification, indicating satisfactory primer specificity. RT-qPCR was subsequently performed using the designed primers and corresponding templates for the 15 candidate genes. Melting curve analysis showed a single peak for each candidate gene (Figure S1), confirming high specificity of the primers and their suitability for subsequent experiments.

### 2.2. Analysis of C_t_ Values of Candidate Reference Genes

**Figure 2:**
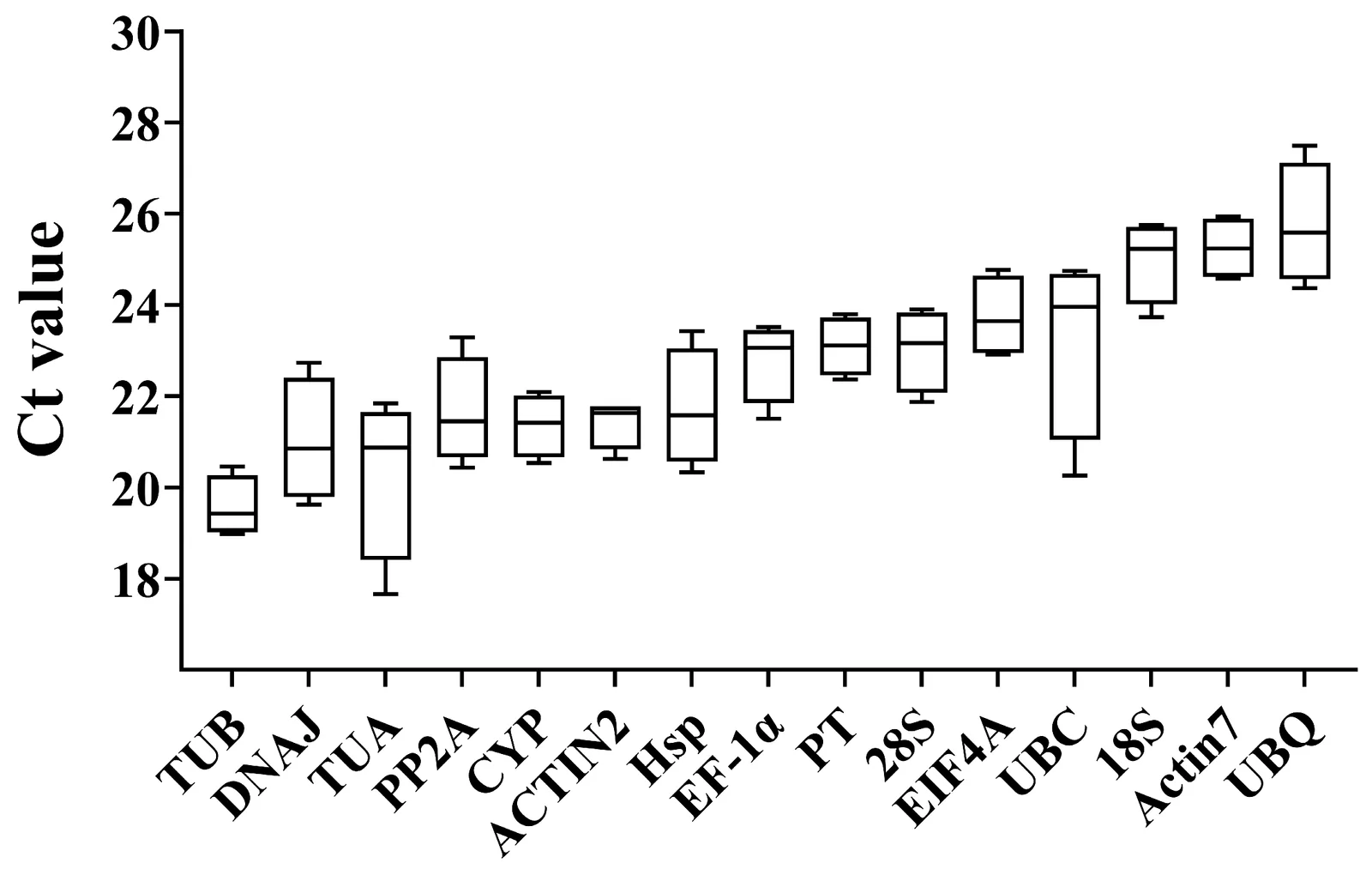
*C_t_* values of 15 candidate reference genes in different organs of *Ilex verticillata*.

The *C_t_* values of the 15 candidate reference genes varied markedly across the four organs of *Ilex verticillata* (Figure 2), with lower *C_t_* values indicating higher transcript abundance. Mean *C_t_* values ranged from 19.57 to 25.76: *TUB* showed the highest average expression level (*C_t_* = 19.57), whereas *UBQ* exhibited the lowest (*C_t_* = 25.76). The extent of *C_t_* fluctuation largely reflects the stability of candidate reference genes. Accordingly, box plots were constructed based on the *C_t_* values of the 15 candidate reference genes. The results revealed that *TUA* and *UBC* exhibited the greatest variability in *C_t_* value—and were thus the least stable—whereas *Actin2* and *Actin7* displayed the narrowest distributions of *C_t_* values and were therefore the most stable.

### 2.3. Stability Analysis of Candidate Reference Genes Across Different Organs of Ilex verticillata

Four statistical algorithms—geNorm, NormFinder, BestKeeper, and RefFinder—were applied to evaluate the expression stability of 15 candidate reference genes across different organs of *Ilex verticillata*, with the aim of preliminarily identifying relatively stable reference genes for subsequent analyses.

**Figure 3:**
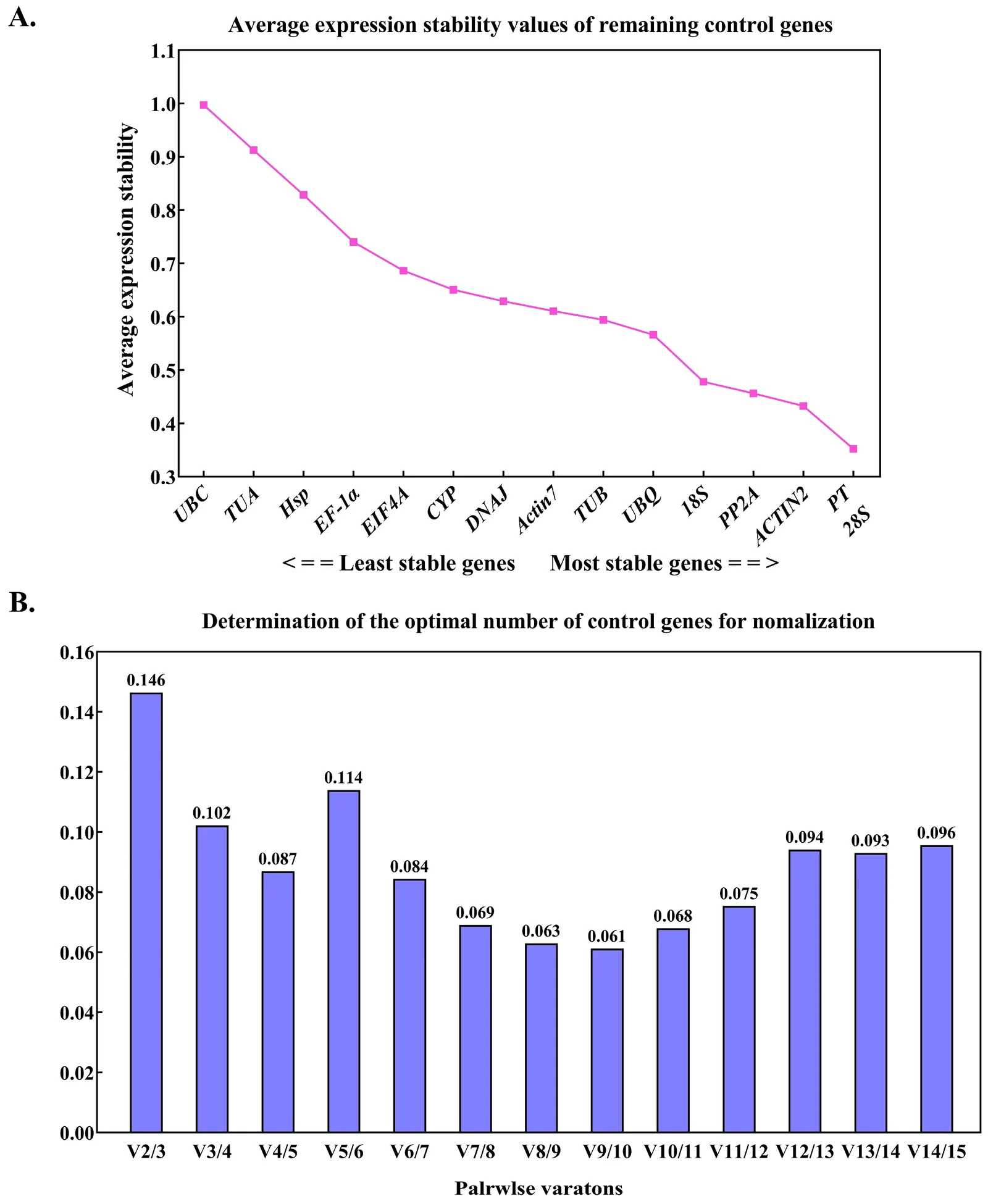
Results of geNorm analysis for the 15 reference genes under different conditions. (**A**) Average expression stability values (M) and ranking of 15 candidate reference genes as calculated by geNorm in different organs of *Ilex verticillata*; (**B**) geNorm analysis of the V values for the 15 reference genes in different organs of *Ilex verticillata*.

The geNorm algorithm evaluates the stability of reference genes by calculating the average expression stability value M, wherein M values below 1.5 indicate stable expression, and a lower M value corresponds to higher stability. In all examined organs of *Ilex verticillata*, the expression stability values (M) of all 15 candidate reference genes were below 1.5, satisfying the widely accepted threshold for suitable reference genes (Figure 3A). Among them, *PT* and *28S* exhibited the lowest M value (0.35), indicating the most stability, whereas *UBC* displayed the highest M value (1.0) and thus the least stability. To optimize data normalization, geNorm recommends the use of two or more reference genes and calculates the pairwise value (*V*) to determine the optimal number of reference genes. The *V_n_*/*V_n_*_+1_ ratio determines the optimal number of reference genes required for reliable normalization. A *V_n_*/*V_n_*_+1_ ratio below 0.15 indicates that *n* reference genes are sufficient; otherwise, *n* + 1 genes are recommended. In this study, the V2/3 ratio was 0.146—below the 0.15 threshold—indicating that the combination of *PT* and *28S* provides adequate stability for normalization across different organs of *Ilex verticillata* (Figure 3B).

Similar to geNorm, NormFinder ranks candidate reference genes based on their calculated stability values, with lower values indicating greater expression stability. In different organs, *PT* and *18S* exhibited the lowest stability values and were thus identified as the most stable and optimal reference genes (Table 1).

BestKeeper assesses the expression stability of candidate reference genes based on the standard deviation (SD), coefficient of variation (CV), and correlation coefficient (*r*) of their *C_t_* values. Among the 15 candidate reference genes, *EIF4A* exhibited the lowest SD value (0.47), indicating the highest expression stability; In contrast, *UBQ*, *EF-1α*, *Actin7*, *TUA*, and *Hsp* displayed SD values exceeding 1.0 and were thus classified as unstable (Table 1).

Because the three algorithms produced somewhat inconsistent rankings, RefFinder was further employed to generate a comprehensive stability ranking (Table 1). According to this integrated analysis, *PT* was identified as the most stable reference gene, followed by *18S*, with stability values (SVs) of 1.86 and 2.34, respectively; In contrast, *TUA* exhibited the lowest stability, with an SV of 14.0. In conjunction with the pairwise variation (*V_n_*/*V_n_*_+1_) values calculated using geNorm, the V_2/3_ value for the *PT* and *18S* combination was below 0.15, confirming that these two genes together provide sufficient stability for normalization across different organs of *Ilex verticillata*.

### 2.4. Stability Analysis of Six Candidate Reference Genes During Flower Bud Differentiation and Hormone Response in Ilex verticillata

Based on organ-level stability analysis, the six top-ranked candidate reference genes identified by RefFinder—*PT*, *18S*, *Actin2*, *28S*, *PP2A*, and *EIF4A*—were selected for further validation. RefFinder was adopted as the primary screening criterion because it integrates and harmonizes the stability rankings generated by three widely used algorithms—geNorm, NormFinder, and BestKeeper—which yielded partially discordant results. The expression stability of these six candidate genes was subsequently evaluated across multiple developmental stages of flower bud formation and under various exogenous hormone treatments. To obtain a robust, system-wide assessment, all expression data were pooled and subjected to comprehensive stability analysis, enabling identification of the most stably expressed reference gene across all experimental conditions.

**Figure 4:**
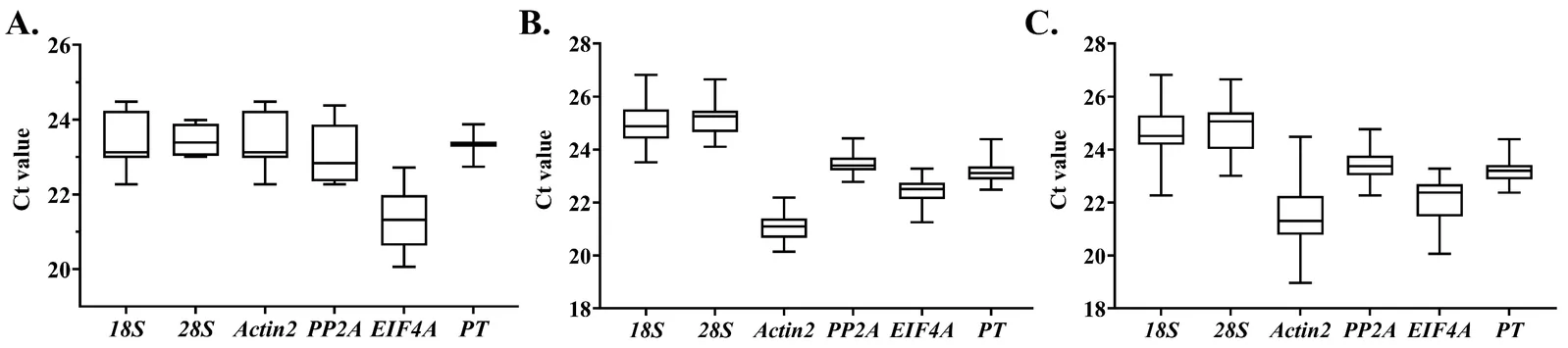
*C_t_* value of 6 candidate reference genes under individual experimental conditions. (**A**) flower buds at different developmental stages, (**B**) different hormone treatments, (**C**) comprehensive tissue and treatments of *Ilex verticillata*.

The *C_t_* values and variation trends of the six candidate reference genes varied among different stages of flower bud differentiation in *Ilex verticillata* (Figure 4A). The results showed that the average *C_t_* values of the six candidate genes ranged from 21.34 to 23.46, with *28S* showing the highest average expression level and *EIF4A* the lowest. Under different hormone treatments (Figure 4B), the average *C_t_* values ranged from 21.07 to 25.17. *Actin2* showed the highest average expression level, and *28S* showed the lowest. Across all samples (Figure 4C), the average *C_t_* values ranged from 21.55 to 24.75, with *Actin2* having the lowest *C_t_* value and *28S* the highest.

**Figure 5:**
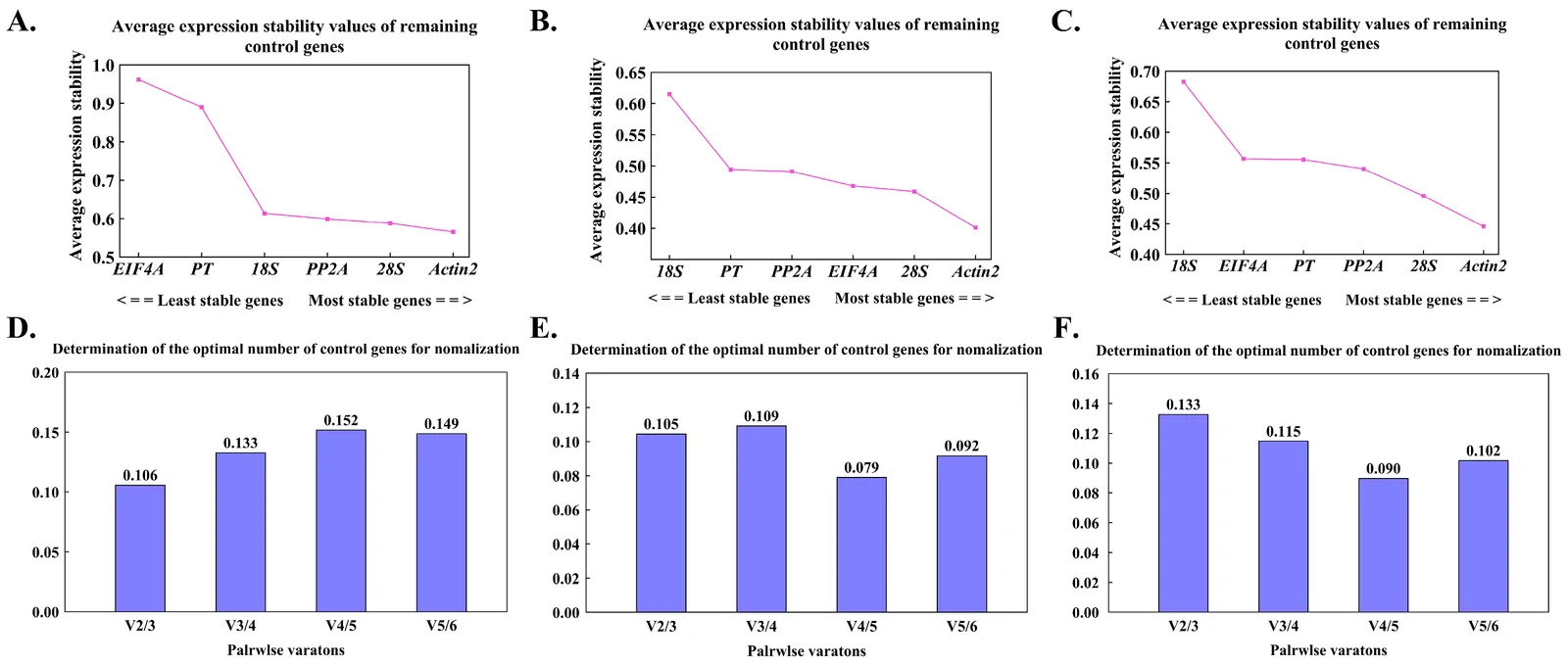
Results of geNorm analysis for the six candidate genes under different conditions. (**A**–**C**) Expression stability of six reference genes under different conditions based on a geNorm analysis; (**D**,**E**) geNorm analysis of the V values for the six reference genes under various conditions; (**A**,**E**) flower buds at different developmental stages, (**B**,**E**) different hormone treatments; (**C**,**F**) all samples.

According to the geNorm analysis, *Actin2* exhibited the lowest M value of 0.565 at different stages of flower bud development, indicating the most stable expression, whereas *EIF4A* showed the poorest expression stability with the highest M value of 0.961 (Figure 5A). Under different hormone treatments, the *28S* and *Actin2* genes exhibited the lowest M values (Figure 5B). In a comprehensive analysis of all sample conditions, *Actin2* and *28S* exhibited the highest stability, with M values of 0.446 and 0.496, respectively, while *18S* showed the poorest stability with an M value of 0.683 (Figure 5C). The pairwise variation values (V) of the normalization factor upon sequential inclusion of additional genes were calculated using geNorm software (Figure 5). The *V_n_*/*V_n_*_+1_ ratios were all below the threshold of 0.15 under all three conditions—during flower bud differentiation, under different hormone treatments, and across all samples combined—being 0.106, 0.105, and 0.133, respectively, indicating that the combination of two reference genes meets the experimental stability requirements.

The NormFinder analysis results (Table 2) showed that *Actin2* exhibited the highest expression stability during flower bud development in *Ilex verticillata*, while *EIF4A* was the least stable. Under different hormone treatments and across all samples combined, *Actin2* was consistently identified as the most stably expressed reference gene. According to the BestKeeper software analysis (Table 3), *PT* was the most stably expressed reference gene at different stages of flower bud differentiation in *Ilex verticillata*. Under different hormone treatments and across all samples combined, *PT* and *PP2A* were the most stably expressed genes, while *18S* remained the most unstable. A comprehensive analysis of the stability of the six reference genes during flower bud differentiation was performed. The RefFinder results (Table 4), which integrate the three software analyses, showed that *Actin2* was the most stably expressed reference gene at different developmental stages of flower buds. *EIF4A* exhibited the poorest expression stability, ranking as the least stable gene in three widely used algorithms (geNorm, NormFinder, and BestKeeper). Under different hormone treatments and across all samples combined, *Actin2* was likewise identified as the most stable gene.

### 2.5. Validation of Reference Gene Stability in Different Organs and During Flower Bud Differentiation of Ilex verticillata

To validate the reliability of the selected reference genes for normalization, we employed both stable reference genes across all experimental conditions and the least stable genes as internal controls to evaluate the expression levels of *IvFT*, *IvAGL19*, and *IvWUS2* in various organs (roots, stems, leaves, and fruits) of *Ilex verticillata*, during flower bud differentiation, and under phytohormone treatment conditions. *IvFT*, *IvAGL19*, and *IvWUS2* are pivotal flowering-regulatory genes that engage in crosstalk with multiple phytohormone signaling pathways, forming a regulatory network that governs floral bud differentiation [15,16,18,19]. Overall, *Actin2*, *PP2A*, and *EIF4A* were identified as relatively stable expressed reference genes across all experimental conditions, whereas 18S was an unstable reference gene.

**Figure 6:**
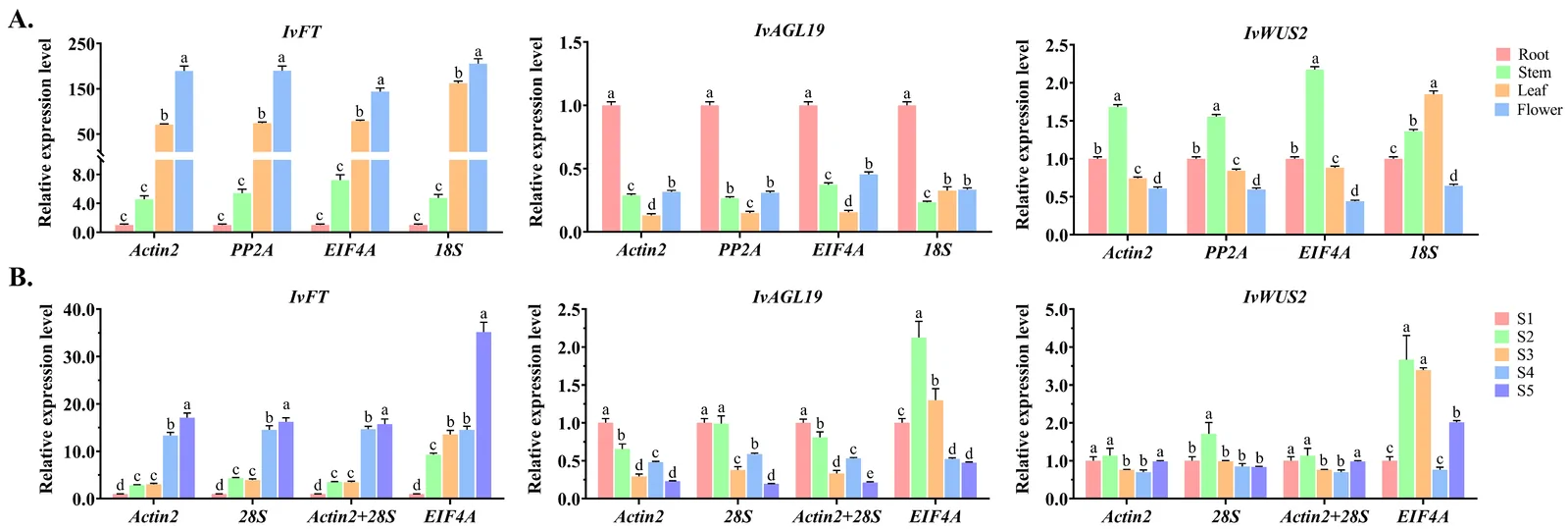
Expression patterns of *IvFT, IvAGL19* and *IvWUS2* genes in different organs (**A**) of *Ilex verticillata* and during flower bud differentiation (**B**), normalized against different reference genes. The different organs are labeled as root, stem, leaf, and flower, respectively. The five developmental stages of flower bud differentiation are designated as S1, S2, S3, S4, and S5. Data are presented as mean ± SD from three biological replicates (*n* = 3). Different lowercase letters indicate significant differences at *p* < 0.05 by one-way ANOVA followed by Duncan’s test.

The expression patterns of *IvFT*, *IvAGL19*, and *IvWUS2* in different organs were shown in Figure 6A. The results demonstrated that when stable reference genes (*Actin2*, *PP2A*, and *EIF4A*) were used for normalization, *IvFT* expression was lowest in roots and highest in flowers, with floral transcript levels approximately 2–3-fold higher than those in leaves. However, when the less stable gene 18S was used as the reference, although *IvFT* still showed the lowest expression in roots and the highest in flowers, the difference between leaves and flowers was markedly reduced. When *Actin2*, *PP2A*, and *EIF4A* were used as internal controls, *IvAGL19* exhibited consistent tissue-specific expression patterns, showing the highest abundance in roots and the lowest in leaves across all organs. However, when 18S was used as the reference gene, the tissue-specific expression pattern of *IvAGL19* differed: although its expression remained highest in roots, it was lowest in stems. When using the most stable genes—*Actin2*, *PP2A*, and *EIF4A*—a consistent expression pattern of the *IvWUS2* gene was observed. In contrast, when the least stable 18S served as the internal control, the tissue-specific expression profile of *IvWUS2* became irregular and lacked any consistent trend.

We further validated the expression patterns of *IvFT*, *IvAGL19*, and *IvWUS2* during flower bud differentiation using the stably expressed reference genes *Actin2* and *28S* (both individually and in combination), and the unstably expressed reference gene *EIF4A*. As shown in Figure 6B, when normalization was performed using the two most suitable reference genes (*Actin2* and *28S*), either alone or in combination, *IvFT* transcript levels increased progressively throughout floral bud differentiation, with comparable expression levels observed between stages S2 and S3, as well as between S4 and S5. In contrast, when the unstable reference gene *EIF4A* was used, *IvFT* expression levels were similar between S3 and S4 but were significantly higher at stage S5 than at stage S4. When *Actin2*, *28S,* or their combination were used as reference genes, *IvAGL19* exhibited the highest transcript level at stage S1, followed by a progressive decline as flower bud development proceeded. *IvWUS2* reached its peak expression at stage S2, remained at relatively low levels during stages S3 and S4, and increased again at stage S5 (Figure 6B). However, when normalization was performed with the unstable reference gene *EIF4A*, the expression pattern of *IvAGL19* changed markedly: *IvAGL19* peaked at stage S2, decreased at stage S3, and reached its lowest levels at stages S4 and S5. Although *IvWUS2* also showed the highest expression at stage S2 under *EIF4A* normalization, it remained at a comparatively high level at stage S3, which was notably different from the results obtained with *Actin2* and *28S*.

### 2.6. Validation of Reference Gene Stability Under Different Hormone Treatments in Ilex verticillata

**Figure 7:**
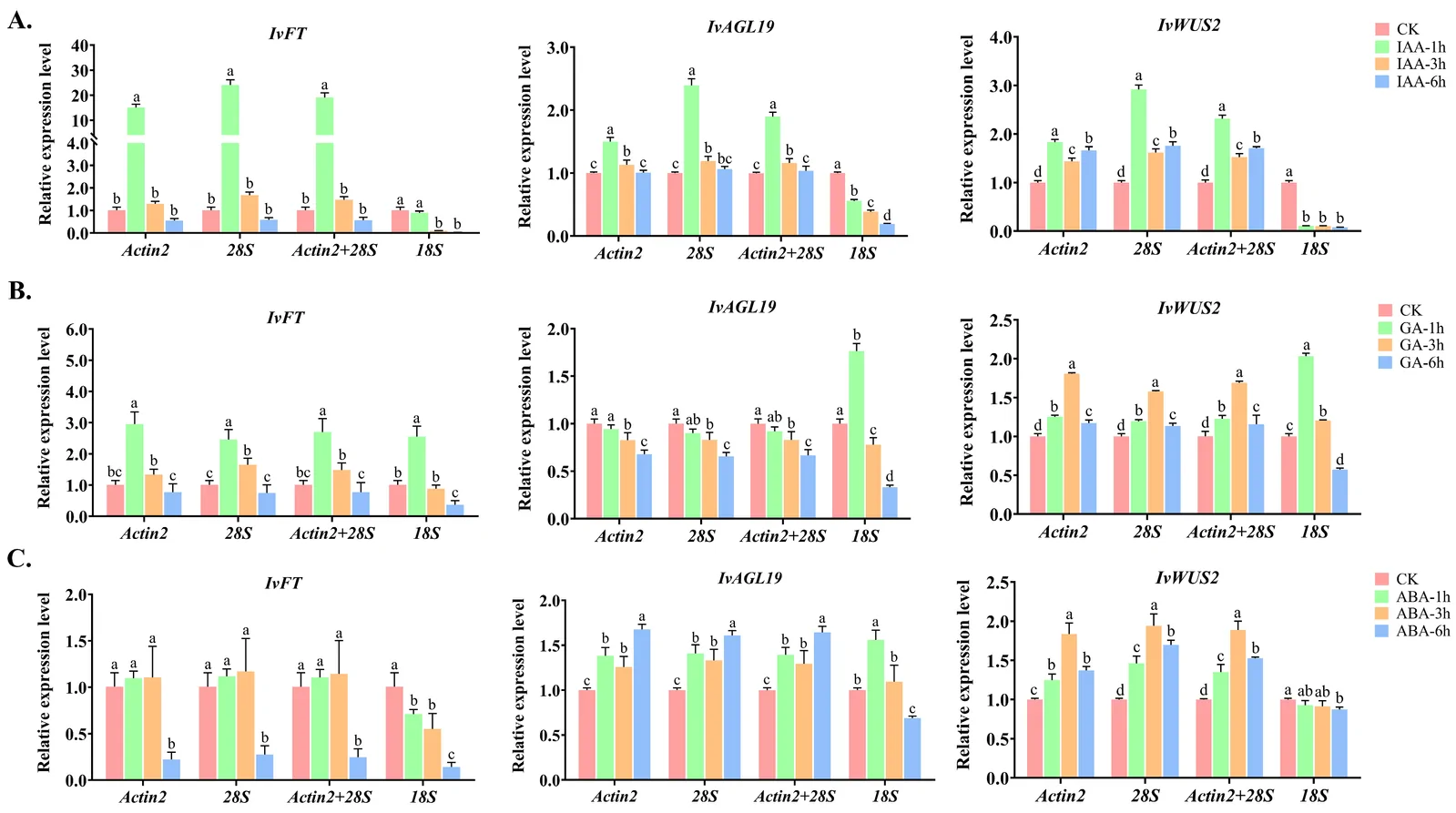
Gene expression patterns of the *IvFT*, *IvAGL19,* and *IvWUS2* genes in *Ilex verticillata* under different hormonal treatment conditions. *Actin2*, *28S*, and *18S* were selected as internal reference genes to determine cycle thresholds; each sample was tested in triplicate. (**A**) Gene expression patterns in the IAA-treated group as determined by real-time quantitative PCR at 0, 1, 3, and 6 h; (**B**) Gene expression patterns in the GA-treated group as determined by real-time quantitative PCR at 0, 1, 3, and 6 h; (**C**) Gene expression patterns in the ABA-treated group as determined by real-time quantitative PCR at 0, 1, 3, and 6 h. Data are presented as mean ± SD from three biological replicates (*n* = 3). Different lowercase letters indicate significant differences at *p* < 0.05 by one-way ANOVA followed by Duncan’s test.

To further evaluate reference gene stability, the stably expressed reference genes (*Actin2* and *28S*, individually and in combination) and the most unstable reference gene (*18S*) were employed to quantify the expression levels of *IvFT*, *IvAGL19*, and *IvWUS2* in response to IAA, GA, and ABA treatments. IAA, GA, and ABA have been reported to be involved in floral bud differentiation, and *IvFT*, *IvAGL19*, and *IvWUS2* are responsive to phytohormone signaling pathways in regulating this process [20,21]. When the two most stable reference genes, either individually or in combination, were used for normalization, the relative expression patterns of *IvFT* exhibited similar trends. In contrast, when the unstable reference gene was employed for relative quantification, the relative expression levels of *IvFT* showed pronounced fluctuations. For example, during the 0–6 h period following IAA treatment, normalization with the stable reference genes (*Actin2* and *28S* or their combination) revealed that *IvFT* expression peaked at 1 h and reached its lowest level at 6 h, showing an overall trend of initial increase followed by a decrease. However, when the least stable gene (*18S*) was used as the reference, *IvFT* exhibited high expression at 0 h that subsequently declined gradually, resulting in a trend markedly different from that observed with the stable references (Figure 7A). Furthermore, in the GA and ABA treatment groups, *Actin2* and *28S* also served as stably expressed reference genes, and the expression levels and trends of *IvFT* exhibited similar patterns across treatments (Figure 7B,C). Likewise, validation of *IvAGL19* and *IvWUS2* using *Actin2*, *28S*, and *18S* yielded the same conclusion: normalization with *Actin2*, *28S,* or their combination resulted in consistent expression patterns under different hormone treatments, whereas normalization with 18S resulted in patterns that differed considerably from those obtained with the stable references. Collectively, these results indicate that the use of inappropriate reference genes may introduce bias in expression analysis. Furthermore, the expression profiles of *IvFT*, *IvWUS2*, and *IvAGL19* demonstrated that the selected reference genes can serve as reliable internal controls for qRT-PCR normalization.

## 3. Discussion

Most species of the genus *Ilex* are evergreen trees with multiple ecological and economic values, including ornamental landscaping, nectar source, timber, medicinal use, and tea production [6,22,23,24,25,26]. *Ilex verticillata* is characterized by abundant red fruits covering the branches after leaf fall in autumn and winter, offering extremely high ornamental value [3,27]. Consequently, this species is widely utilized in landscape gardening, potted ornamentals, and high-grade fresh cut flower production [6]. To date, research on *Ilex verticillata* has mostly focused on conventional breeding and cultivation physiology, whereas molecular mechanism studies related to its growth and development remain insufficient [4,5,28]. RT-qPCR is an indispensable technical approach for quantifying gene expression across different organs, developmental stages, and under external stresses [29]. Reliable reference genes are essential for accurate normalization, but no universal reference gene exists for all species and all experimental conditions [30,31,32,33,34,35]. Although reference gene validation has been systematically performed in a wide range of plant species, no relevant study reporting stable candidate reference genes for *Ilex verticillata* has been reported [36,37,38,39].

In this study, 15 candidate reference genes, namely *18S*, *28S*, *CYP*, *DNAJ*, *EF-1α*, *Hsp*, *EIF4A*, *PP2A*, *PT*, *TUA*, *TUB*, *UBC*, *UBQ*, *Actin2,* and *Actin7*, were selected based on transcriptome data and existing literature (Table S2) [12,35,40,41,42,43]. The *C_t_* value is defined as the cycle number at which the fluorescence signal exceeds the preset threshold, and it serves as the fundamental parameter for quantitative analysis in real-time quantitative PCR. In general, *C_t_* values between 15 and 35 represent a reliable range for data interpretation (Figure 2) [8,44]. The *C_t_* values of all candidate genes across different organs ranged from 19.57 to 25.76, which fell within the acceptable quantitative range. Among these candidates, *TUB* exhibited the lowest average *C_t_* value (19.57), while *UBQ* showed the highest (25.76), suggesting considerable variation in basal expression of candidate genes amonsg different organs. The gene stability rankings generated by geNorm, NormFinder, and BestKeeper differed considerably (Figure 3) (Table 1). geNorm analysis indicated that *PT* and *28S* had the lowest M values and were the most stable genes across different organs. According to NormFinder, *PT* and *18S* were the most stable candidate genes. Compared with the results of geNorm and NormFinder, BestKeeper identified *EIF4A* as the most stable candidate gene. Such discrepancies mainly arise from the different statistical principles underlying these software tools. geNorm evaluates the average pairwise variation among candidate genes and tends to select genes with similar expression patterns across all samples [10]. NormFinder estimates intra- and inter-group variation, yielding more accurate results in subsets composed of different sample types [45]. BestKeeper identifies the most stable reference genes through repeated pairwise correlation analysis, and its results often differ from those of geNorm and NormFinder [46]. Since no single algorithm has been confirmed as universally superior for reference gene evaluation, comprehensive integration of multiple analytical tools is essential. In this study, RefFinder was used to integrate the ranking results from geNorm, NormFinder, and BestKeeper and generate a comprehensive stability profile for all candidate reference genes [47]. The comprehensive ranking generated by RefFinder showed that *PT* exhibited the highest stability, followed by *18S*, *Actin2*, *28S*, *PP2A*, and *EIF4A* (Table 1). Therefore, these six genes were selected as preferred candidate reference genes for stability validation.

As a prominent ornamental fruiting landscape plant, the flower bud differentiation and germination of *Ilex verticillata* are critical biological processes that determine its ornamental traits and landscape value. The temporal progression of flower bud development is coordinately regulated by multiple plant hormones [27,48,49]. For samples collected at different flower bud development stages, both geNorm and NormFinder identified *Actin2* as the most stable gene, while RefFinder ranked *Actin2* as the top-ranked gene, with *28S* ranked second (Figure 5; Table 2 and Table 4). Under hormone treatments, geNorm ranked 28S and Actin2 as the most stable genes, while NormFinder and RefFinder both prioritized *Actin2* first, whereas BestKeeper prioritized *PT* and *PP2A* as the most stable candidates (Table 2, Table 3 and Table 4). In addition, *18S* was classified as the least stable gene under hormone treatments. Across all samples, *Actin2* displayed the highest comprehensive stability, followed by *PP2A* and *28S*, whereas *18S* showed the least stability. In conventional RT-qPCR experiments, normalization using a single reference gene is generally insufficient to ensure reliable results. The geNorm algorithm can determine the optimal number of reference genes by calculating pairwise variation values. In this study, all pairwise variation values *V_n_*/*V_n_*_+1_ were below 0.15 under different organs, flower bud differentiation, and hormone treatments, indicating that two reference genes were sufficient for reliable normalization in *Ilex verticillata*. Integrated analysis of flower bud differentiation, hormone response, and all pooled samples consistently demonstrated that the combination of *Actin2* and *28S* represented the optimal reference gene pair.

*Actin* is a classical housekeeping gene widely used for plant gene expression normalization, yet its stability is highly dependent on plant species and conditions [50,51]. For example, *ACT* was unstable during navel orange fruit ripening [52], *ACT1* shows the lowest stability across different developmental stages of *Allium wallichii* [41], and β-actin (*ACTB*) showed the poorest stability in *Tagetes erecta* [35]. In hormone-treated organs of *Scutellaria baicalensis*, *ACT7* is found to be the most stable reference gene, whereas *ACT11* is highly unstable. These findings illustrate that homologous genes with similar sequences, structures, and functions may exhibit distinct expression levels and stability profiles. As a high-abundance ribosomal RNA gene, *28S* rRNA has been widely employed for gene normalization in plant development and stress responses. For example, *28S* and *EF1α* were identified as the optimal reference genes during *Avena fatua* development [53], and *28S* exhibits superior stability under cold stress in *Michelia compressa* [54]. In this study, *28S* maintained high stability across different organs, flower bud differentiation, and hormone-response conditions, and the combination of *Actin2* and 28S was validated as the optimal reference gene pair. Overall, these results indicate that the *Actin2* + *28S* combination is reliable for RT-qPCR normalization in studies of flower bud differentiation and hormone response in *Ilex verticillata*, and further indicate that reference gene selection should be optimized for different biological samples.

Finally, three flowering-related target genes—*IvFT*, *IvAGL19*, and *IvWUS2*—were used to validate the stability of the identified candidate reference genes across different organs, during flower bud differentiation, and under phytohormone treatments. *FT* encodes florigen and regulates floral induction; *AGL19* is a MADS-box transcription factor that promotes flowering; and *WUS* is a homeodomain transcription factor involved in meristem stem cell maintenance and cytokinin signaling. These three genes represent distinct regulatory levels of flowering and are closely linked to phytohormone signaling. Normalization with the most stable individual genes (*Actin2* and *28S*) or their optimal combination (*Actin2* + *28S*) yielded consistent and reproducible expression patterns for the target genes. In contrast, normalization using the least stable reference genes (*18S* or *EIF4A*) produced inconsistent and distorted expression profiles. These results further confirm the reliability of *Actin2* and *28S* and emphasize that appropriate reference gene selection is a prerequisite for credible RT-qPCR quantification.

In summary, this study provides the first systematic validation of reference genes for RT-qPCR in *Ilex verticillata*. Among all tested candidates, *Actin2* exhibited the highest expression stability across different organs, floral bud developmental stages, and phytohormone treatments. When a two-gene normalization strategy is preferred, the combination of *Actin2* and *28S* is recommended as the optimal reference gene pair. These findings provide a valuable resource for future investigations on flower bud differentiation, hormone signaling, and flowering regulatory mechanisms in *Ilex verticillata*.

## 4. Materials and Methods

### 4.1. Experimental Materials

The experimental materials were obtained from the Fruit Tree Garden of Zhejiang A&F University (119°43′42″ E, 30°15′16″ N). Two-year-old *Ilex verticillata* plants with vigorous growth were used as test materials. Samples from distinct organs, including roots, stems, leaves, and fruits, as well as flower buds at different developmental stages, were harvested.

For phytohormone treatments, two-year-old *Ilex verticillata* plants with consistent growth were similarly selected. Nine phytohormones were applied as foliar sprays: including indole-3-acetic acid (IAA, 100 μmol·L^−1^), indole-3-butyric acid (IBA, 100 μmol·L^−1^), N-(1-naphthyl) phthalamic acid (NPA, 10 μmol·L^−1^), kinetin (KT, 100 μmol·L^−1^), abscisic acid (ABA, 100 μmol·L^−1^), salicylic acid (SA, 200 μmol·L^−1^), gibberellic acid (GA_3_, 200 μmol·L^−1^), ethephon (ACC, 1 mmol·L^−1^), and methyl jasmonate (MeJA, 100 μmol·L^−1^), with distilled water serving as the control. Spraying was continued until both the upper and lower leaf surfaces were evenly wetted. Leaves of *Ilex verticillata* were sampled at 0, 1, 3, 6, and 12 h after treatment. Three biological replicates were obtained for each sample. Immediately after collection, samples were snap-frozen in liquid nitrogen and subsequently stored at −80 °C until further use.

### 4.2. Total RNA Extraction and cDNA Synthesis

RNA was extracted from each sample using the RNAprep Pure Polysaccharide–Polyphenol Plant Total RNA Extraction Kit (DP441, TianGen Biotech, Beijing, China), strictly following the manufacturer’s instructions. RNA integrity was assessed by electrophoresis on a 1.2% agarose gel, and RNA concentration and purity were quantified using a Thermo Scientific (NanoDrop 1000 spectrophotometer, Wilmington, DE, USA). First-strand cDNA was subsequently synthesized using the PrimerScript™ RT Master Mix (Perfect Real Time) (RR036A, Takara, Dalian, China), following the manufacturer’s instructions. The resulting cDNA was stored at −20 °C until further use.

### 4.3. Selection of Candidate Reference Genes and Primer Design

Fifteen candidate reference genes were selected based on prior studies on reference gene validation and *Ilex verticillata* transcriptome sequencing data. These genes include: *18S* (18S ribosomal RNA), *28S* (28S ribosomal RNA), *CYP* (Cyclophilin), *DNAJ* (DnaJ heat shock protein), *EF-1α* (elongation factor 1-α), *Hsp* (heat shock protein), *EIF4A* (eukaryotic initiation factor 4A), *PP2A* (protein phosphatase 2A regulatory subunit A), *PT* (Potassium transporter-like), *TUA* (α-tubulin), *TUB* (β-tubulin), UBC (ubiquitin-conjugating enzyme), *UBQ* (ubiquitin), *Actin2* (β-Actin-2), and *Actin7* (β-Actin-7) [12,35,40,41,42,43]. Gene-specific RT-qPCR primers were designed using Primer 5 software (Table S2) with the following criteria: GC content of 45–65%, optimal annealing temperature of 55–65 °C, primer length of 15–25 bp, and amplicon size of 110–220 bp. All primers were synthesized by Sangon Biotech (Shanghai, China). Preliminary primer screening was conducted via conventional PCR followed by agarose gel electrophoresis. Only primers yielding a single, sharp band of expected size—without nonspecific amplification or primer-dimer formation—were retained. Subsequently, melting-curve analysis was performed to verify primer specificity; only primer pairs exhibiting a single, well-defined melting peak were selected for subsequent RT-qPCR experiments.

### 4.4. Quantitative RT-PCR Analysis

RT-qPCR was performed on a BIO-RAD CFX96 real-time PCR system. Each 10 μL amplification mixture consisted of 5 μL TB Green Taq (2×), 2 μL nuclease-free H_2_O, 0.5 μL forward primer, 0.5 μL reverse primer (both at 10 μmol·L^−1^), and 2 μL diluted cDNA template. The amplification protocol started with pre-denaturation at 95 °C for 30 s, followed by 40 cycles of denaturation at 95 °C for 5 s and annealing/extension at 60 °C for 30 s. Subsequently, melting curve analysis was conducted from 55 °C to 95 °C to confirm amplification specificity. For each experimental condition, three independent biological replicates were analyzed, with each biological replicate comprising three technical replicates. Data acquisition and analysis were performed using BioRad CFX Manager (v3.1) software.

### 4.5. Data Analysis

The *C_t_*/*C_q_* value is inversely correlated with transcript abundance: a lower *C_t_*/*C_q_* value indicates higher gene expression. Four complementary algorithms—geNorm, NormFinder, BestKeeper, and RefFinder—were employed to assess and rank the expression stability of candidate reference genes [10,45,46,47]. For geNorm (https://seqyuan.shinyapps.io/seqyuan_prosper/, accessed on 13 September 2025) analysis, raw *C_t_* values were first transformed into relative expression levels using the 2^−Δ*Ct*^ method, where *ΔC_t_* was calculated as the difference between the *C_t_* values of each sample and the minimum *C_t_* values observed across the entire dataset. These normalized expression values were then imported into the geNorm (v3.5) software [10]. Within geNorm, the expression stability measure (M) is computed for each candidate reference gene; a lower M value indicates higher expression stability, and a threshold of M = 1.5 is applied to assess suitability—genes with M > 1.5 are considered highly variable and thus excluded from further consideration as reference genes. Furthermore, geNorm calculates pairwise variation (V) values by comparing successive normalization factors generated through the stepwise inclusion of additional candidate genes. The *V_n_*/*V_n_*_+1_ ratio is subsequently employed to determine the minimum number of reference genes required for reliable normalization: when this ratio falls below 0.15, *n* reference genes are deemed sufficient. NormFinder (https://seqyuan.shinyapps.io/seqyuan_prosper/, accessed on 13 September 2025) first converts raw *C_t_* values into relative expression quantities and then partitions the total variation into intra-group and inter-group components, thereby deriving a stability measure (M) for each candidate gene [45]. As in geNorm, a lower *M* value reflects greater expression stability. BestKeeper (https://www.gene-quantification.de/bestkeeper.html, accessed on 13 September 2025) assesses gene stability based on three descriptive statistics calculated directly from *C_t_* values: the standard deviation (SD), coefficient of variation (CV), and correlation coefficient (*r*) across samples [46]. Lower SD and CV values indicate higher expression stability. Notably, an SD > 1 is considered indicative of unacceptable variability; such genes are therefore excluded from further consideration as reference genes. RefFinder (https://blooge.cn/RefFinder/?type=reference, accessed on 13 September 2025) integrates the rankings generated by the three aforementioned algorithms by assigning a weighted score to each candidate gene and computing the geometric mean of these scores, yielding a comprehensive, consensus-based stability ranking [47].

## 5. Conclusions

In summary, this study systematically identified stably expressed reference genes for RT-qPCR normalization in *Ilex verticillata* across multiple experimental conditions. The expression stability of 15 candidate reference genes was first evaluated across various plant organs using complementary algorithms—geNorm, NormFinder, BestKeeper, and RefFinder. Six genes—*PT*, *18S*, *Actin2*, *28S*, *PP2A*, and *EIF4A*—demonstrated superior stability and were therefore selected for further rigorous validation across successive stages of flower bud differentiation and under exogenous hormone treatments. Collectively, *Actin2* and *28S* are recommended as the optimal reference genes for gene expression studies related to flower bud differentiation and plant hormone signaling in *Ilex verticillata*. These findings not only enhance the accuracy and reliability of RT-qPCR-based quantification of target gene expression but also establish a robust methodological foundation for further investigations into the molecular mechanisms underlying flower bud differentiation in *Ilex verticillata*, with potential translational relevance to other species within the genus *Ilex*.

## Figures and Tables

**Table 1:** Expression stability of candidate reference genes in different organs of *Ilex verticillata* calculated by NormFinder, BestKeeper, and RefFinder.

Rank	NormFinder		BestKeeper				RefFinder	
	Gene	SV	Gene	SD	CV	*r*	Gene	SV
1	*18S*	0.151	*EIF4A*	0.47	2.19	0.52	*PT*	1.86
2	*PT*	0.212	*UBC*	0.58	2.70	0.297	*18S*	2.34
3	*Actin2*	0.246	*PT*	0.59	2.55	0.935	*Actin2*	3.22
4	*DNAJ*	0.296	*Actin2*	0.67	3.45	0.874	*28S*	3.5
5	*PP2A*	0.315	*28S*	0.73	2.90	0.834	*PP2A*	5.18
6	*28S*	0.348	*18S*	0.83	3.33	0.966	*EIF4A*	6.04
7	*CYP*	0.352	*DNAJ*	0.87	3.76	0.917	*DNAJ*	6.24
8	*UBQ*	0.377	*TUB*	0.91	4.21	0.93	*UBQ*	7.8
9	*TUB*	0.387	*PP2A*	0.95	4.00	0.929	*TUB*	8.21
10	*Actin7*	0.439	*CYP*	0.96	4.21	0.909	*CYP*	8.65
11	*EIF4A*	0.557	*UBQ*	1.01	4.66	0.891	*UBC*	9.06
12	*EF-1α*	0.625	*EF-1α*	1.10	5.24	0.786	*Actin7*	10.1
13	*Hsp*	0.837	*Actin7*	1.17	4.55	0.905	*EF-1α*	12
14	*TUA*	0.892	*TUA*	1.39	6.85	0.745	*Hsp*	13.47
15	*UBC*	0.986	*Hsp*	1.56	6.71	0.889	*TUA*	14

Note: SV, stability value. For NormFinder and RefFinder, a lower SV indicates higher expression stability. For BestKeeper, SD and CV were used to evaluate stability; a lower SD or CV indicates higher stability. *r*, correlation coefficient.

**Table 2:** Analysis of expression stability of six candidate reference genes of *Ilex verticillata* under individual experimental conditions by NormFinder.

Rank	Flower		Hormonal Treatment		All Samples	
	Gene	SV	Gene	SV	Gene	SV
1	*Actin2*	0.155	*Actin2*	0.112	*Actin2*	0.110
2	*18S*	0.179	*28S*	0.211	*28S*	0.212
3	*28S*	0.218	*EIF4A*	0.218	*PP2A*	0.260
4	*PP2A*	0.236	*PP2A*	0.241	*PT*	0.279
5	*PT*	0.549	*PT*	0.251	*EIF4A*	0.285
6	*EIF4A*	0.603	*18S*	0.370	*18S*	0.410

Note: SV, stability value calculated by NormFinder. A lower SV indicates higher expression stability.

**Table 3:** Analysis of expression stability of six candidate internal reference genes of *Ilex verticillata* under individual experimental conditions by BestKeeper.

Rank	Flower		Hormonal Treatment		All Samples	
	Gene	SD	Gene	SD	Gene	SD
1	*PT*	0.22	*PT*	0.32	*PT*	0.33
2	*18S*	0.35	*PP2A*	0.32	*PP2A*	0.41
3	*Actin2*	0.65	*Actin2*	0.37	*Actin2*	0.54
4	*28S*	0.72	*EIF4A*	0.45	*EIF4A*	0.60
5	*EIF4A*	0.76	*28S*	0.46	*28S*	0.63
6	*PP2A*	0.76	*18S*	0.63	*18S*	0.71

Note: SD, standard deviation, calculated by BestKeeper. A lower SD indicates higher expression stability.

**Table 4:** Analysis of expression stability of six candidate internal reference genes of *Ilex verticillata* under individual experimental conditions by RefFinder.

Rank	Flower		Hormonal Treatment		All Samples	
	Gene	SV	Gene	SV	Gene	SV
1	*Actin2*	1.73	*Actin2*	1.32	*Actin2*	1.32
2	*28S*	2.21	*28S*	2.11	*PP2A*	2.38
3	*PP2A*	2.78	*PT*	3.16	*EIF4A*	2.45
4	*18S*	2.83	*EIF4A*	3.22	*PT*	3.34
5	*PT*	3.34	*PP2A*	3.56	*28S*	3.94
6	*EIF4A*	6.00	*18S*	6.00	*18S*	6.00

Note: SV, comprehensive stability value, calculated by RefFinder. A lower SV indicates higher expression stability.

## Data Availability

The original contributions presented in this study are included in the article. Further inquiries can be directed to the corresponding authors.

## Supplementary Materials

The following supporting information can be downloaded at: https://www.mdpi.com/article/10.3390/plants15193001/s1. Figure S1: Melting curves illustrating primer specificity for candidate housekeeping genes; Table S1: Reference gene sequences; Table S2: Primer information of 15 candidate genes.
