# Supplementary material for: Selection and Validation of Reference Genes for RT-qPCR Across Different Organs, Flower Bud Developmental Stages, and Hormone Treatments in Ilex verticillata
Source: Plants (Basel). 2026 Sep 30;15(19):3001. doi: 10.3390/plants15193001 (PMC13666670; doi:10.3390/plants15193001)
Supplement: Supplementary file 1 [file plants-15-03001-s001.zip › Supplementary Figure S1.pdf]

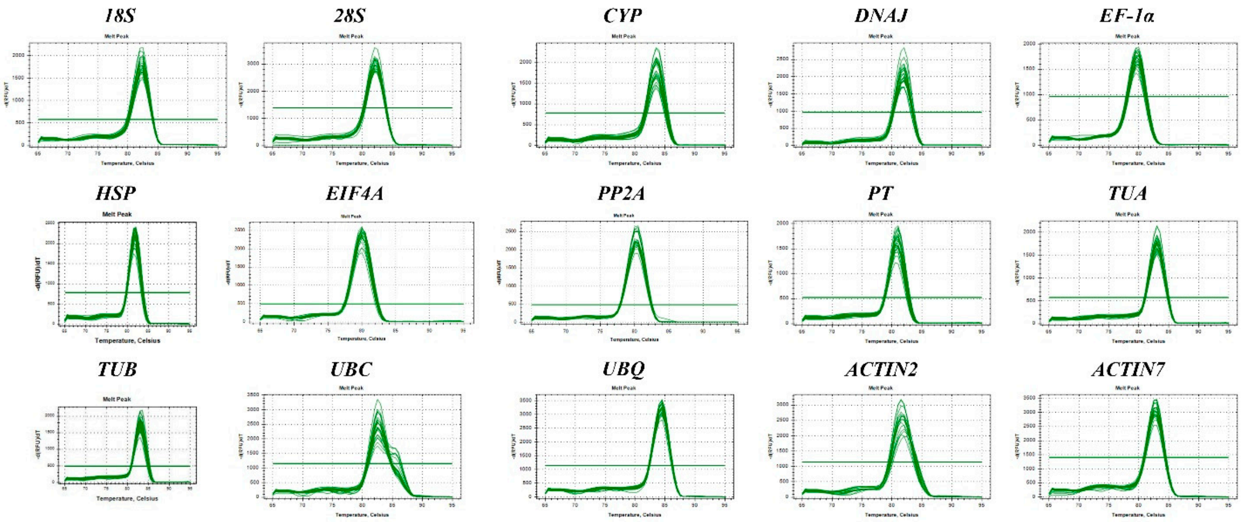

FigureS1 Melting curves illustrating the specificity of primers for candidate housekeeping genes
