# Supplementary material for: Selection and Validation of Reference Genes for RT-qPCR Across Different Organs, Flower Bud Developmental Stages, and Hormone Treatments in Ilex verticillata
Source: Plants (Basel). 2026 Sep 30;15(19):3001. doi: 10.3390/plants15193001 (PMC13666670; doi:10.3390/plants15193001)
Supplement: Supplementary file 1 [file plants-15-03001-s001.zip › Supplementary Table S2.pdf]

Table S2 Primer information of 15 candidate genes.

Gene	Full name of the gene	Primer sequence	Tm/℃	Product length
18S	18S ribosomal RNA	F: AGATGTTTGGCAAGGGGAGC	61.4	174 bp
		R: TGGACCACAAGTTAGCACAAGG	60.2	
28S	28S ribosomal RNA	F: AAGTGCAAGCTCTTTTCTGCC	59.2	154 bp
		R: CGCCTCTTTCATTTCATTCG	58.5	
CYP	Cyclophilin	F: CCCTATCCACTATCGCAACCG	62	128 bp
		R: GGCTCCTGCTACGAGAAGATG	59.2	
DNAJ	DnaJ heat shock protein	F: TACAGAAAGGCATTGGCACG	59.6	183 bp
		R: ACCAGGAACTTTGCTGCTACTC	58.2	
EF-1α	Eukaryotic elongation faCtor 1-*α*	F: CAAACCCCTTCGTCTCCCA	60.4	110 bp
		R: TCAACACGTCCTACTGGCACA	58.5	
Hsp	Heat shock protein	F: TCGGTCCCTTCCAATCAACA	60.8	119 bp
		R: TTCAGCATCCTCTTCTTCCAAA	59.3	
EIF4A	ukaryotic initiation faCtor 4A	F: AGGATTTTGGTGAAACGTGATG	59.2	116 bp
		R: GCCAGGGTCTCATAGAGGTCA	58.9	
PP2A	protein phosphatase 2A regulatory subunit A	F: GCCGTGACTACATGACCCTTA	57.6	173 bp
		R: AGTTGCTACTCGCAGACCCTC	68.7	
PT	Potassium transporter like	F: TTCCAGTACCCTATGTCTCCCC	59.7	132 bp
		R: TCTCAAAGTTCCCATCATCTCG	59.3	
TUA	a-tubulin	F: TTGATGGAGCCTTGAATGTCG	60.9	205 bp
		R: TATTTGCCTTGGCGTGGG	60	
TUB	β-tubulin	F: TGGACAATGAGGCTCTGTATGA	58	121 bp
		R: AAGCGGAGGCAGCAAGTCA	61.5	
UBC	ubiquitin-conjugating enzyme	F: TACTCCAGTTGCCATCTCCATT	58.9	155 bp
		R: AACCACGCCACCTTCCTTC	59.6	
UBQ	Polyubiquitin	F: AAAACAATCACTCTGGAGGTCG	58.5	193 bp
		R: CCCTCAATCGCAGAACCAA	59	
Actin2	β-Actin-2	F: GAAATCACTGCCCTTGCTCC	59.5	122 bp
		R: CACATCTGTTGGAAAGTGCTGA	58.6	
Actin7	β-Actin-7	F: GAAGTGTTTGAAGACTCCCTCG	58.2	108 bp
		R: TGGCACCTTAATCTTCTTCTCG	59.1	
